# Identifying Drivers of Overall Satisfaction in Patients Receiving HIV Primary Care: A Cross-Sectional Study

**DOI:** 10.1371/journal.pone.0042980

**Published:** 2012-08-13

**Authors:** Bich N. Dang, Robert A. Westbrook, Maria C. Rodriguez-Barradas, Thomas P. Giordano

**Affiliations:** 1 Houston Veterans Affairs Health Services Research and Development Center of Excellence, Houston, Texas, United States of America; 2 Michael E. DeBakey Veterans Affairs Medical Center, Houston, Texas, United States of America; 3 Department of Medicine, Baylor College of Medicine, Houston, Texas, United States of America; 4 Harris County Hospital District, Houston, Texas, United States of America; 5 Jesse H. Jones Graduate School of Business, Rice University, Houston, Texas, United States of America; The James Cook University Hospital, United Kingdom

## Abstract

**Objective:**

This study seeks to understand the drivers of overall patient satisfaction in a predominantly low-income, ethnic-minority population of HIV primary care patients. The study’s primary aims were to determine 1) the component experiences which contribute to patients’ evaluations of their overall satisfaction with care received, and 2) the relative contribution of each component experience in explaining patients’ evaluation of overall satisfaction.

**Methods:**

We conducted a cross-sectional study of 489 adult patients receiving HIV primary care at two clinics in Houston, Texas, from January 13–April 21, 2011. The participation rate among eligible patients was 94%. The survey included 15 questions about various components of the care experience, 4 questions about the provider experience and 3 questions about overall care. To ensure that the survey was appropriately tailored to our clinic population and the list of component experiences reflected all aspects of the care experience salient to patients, we conducted in-depth interviews with key providers and clinic staff and pre-tested the survey instrument with patients.

**Results:**

Patients’ evaluation of their provider correlated the strongest with their overall satisfaction (standardized β = 0.445, p<0.001) and accounted for almost half of the explained variance. Access and availability, like clinic hours and ease of calling the clinic, also correlated with overall satisfaction, but less strongly. Wait time and parking, despite receiving low patient ratings, did not correlate with overall satisfaction.

**Conclusions:**

The patient-provider relationship far exceeds other component experiences of care in its association with overall satisfaction. Our study suggests that interventions to improve overall patient satisfaction should focus on improving patients’ evaluation of their provider.

## Introduction

The use of self-reported patient evaluations as a quality measure signifies a paradigm shift in American medicine. The Consumer Assessment of Healthcare Providers and Systems (CAHPS®) Hospital Survey, developed by the Centers for Medicare and Medicaid Services (CMS) and the Agency for Healthcare Research and Quality, represents the first standardized, nationwide measurement system for tracking patients’ perception of their care [1]. CMS reports CAHPS® Hospital Survey results publicly, and starting October 2012, Medicare will distribute value-based incentive payments for acute care services based partly on how patients rate their care experience [2]. In addition, the American Board of Internal Medicine requires recertifying physicians to complete at least one Practice Improvement Module®, one of which entails soliciting 25 patient evaluation surveys.

The focus on patient satisfaction stems from longstanding interest in the business sector, where most large firms regularly monitor the satisfaction of their customers. The emphasis on improving customer experience is based on evidence that higher levels of customer satisfaction lead to higher customer loyalty, greater repeat purchasing and more favorable referrals, all of which result in improved market share, greater revenues and higher profitability [3]. Customer satisfaction serves as a key metric for judging firm performance and informs management on how to improve customer experiences with their firm’s offerings and sales channels.

In the context of health care, patient satisfaction is an individual’s evaluation of his or her experiences in receiving health care in a specific delivery setting (e.g. hospital, primary care clinic, outpatient surgery, etc) [4]. Limited cross-sectional studies show a positive relationship between patient satisfaction and adherence to medications [5]–[9]. Likewise, adherence to medications clearly impacts clinical outcomes [10], [11]. Satisfaction also has been associated with patient switching behavior in regards to provider and insurance plans [12], [13]. Furthermore, studies using national CAHPS® Hospital Survey data show a significant albeit modest correlation between patient satisfaction and objective clinical performance measures [14], [15].

Leaders seeking to improve satisfaction typically apply the attribute model of satisfaction [16], [17]. Attribute models have been used to study satisfaction across a wide spectrum of human experience, including customer, job and life satisfaction [18], [19]. In the context of health care, this model incorporates the following concepts: 1) overall patient satisfaction describes a distinct and separate global evaluation of a set of component experiences; 2) given a set of experiences, patients weigh each experience differently in rating their overall satisfaction; 3) the stronger the association between a component experience and overall satisfaction, the greater the presumed impact of that component experience. The attribute model of satisfaction provides insight into the relative importance of different component experiences to patients and the trade-offs patients may make in exchange for excellence in other areas. Furthermore, component experiences attributed the most importance represent critical points of intervention for improving the care experience and ultimately increasing overall satisfaction.

Relatively fewer patient satisfaction studies take place in the outpatient primary care setting and in the context of chronic diseases. While the CAHPS® database of national hospital survey data provides a glimpse of patients’ hospital care experience, CMS does not require the public reporting of outpatient clinic survey data. HIV affects over 1.1 million people in the United States and represents a chronic disease well-suited for studying the drivers of overall patient satisfaction [20]. Management of HIV infection occurs mostly in the outpatient setting and requires frequent visits with a primary care provider. In this study, we seek to understand the drivers of overall patient satisfaction in a predominantly low-income, ethnic-minority population of HIV primary care patients, a group not well-represented in patient satisfaction studies. Specifically, we applied the attribute model of satisfaction to determine 1) the component experiences that contribute to patients’ evaluation of their overall satisfaction with care received, and 2) the relative contribution of each component experience in explaining patients’ evaluation of overall satisfaction.

## Methods

### Subjects

We conducted a cross-sectional study of patients receiving outpatient HIV primary care at Thomas Street Health Center (TSHC) and the Michael E. DeBakey Veterans Affairs Medical Center (VAMC) in Houston, Texas. Patients enrolled in the study from January 13 to April 21, 2011. Inclusion criteria included: 1) age 18 years or older; 2) having at least one HIV primary care visit in the past year; and 3) having an “index” visit at least one year prior to enrollment. An “index” visit was defined as an HIV primary care visit with a doctor, advanced nurse practitioner, or physician assistant. Exclusion criteria included: 1) incarceration >30 days in the past year; 2) mental or physical inability to complete the survey; and 3) inability to complete the survey in English or Spanish. These criteria ensured that patients had sufficient exposure to the clinic to assess their overall satisfaction.

### Survey Instrument

Satisfaction questions were adapted from validated patient self-report survey instruments. The survey measured patients’ evaluations of their component experiences and overall satisfaction with care in clinic. Questions measured cumulative satisfaction over the most recent 12-month time frame. The survey included 15 questions about various components of the care experience, 4 questions about the provider experience, and 3 questions about the overall care experience. Provider referred to a doctor, nurse practitioner or physician assistant. Table 1 shows the exact wording of the items.

**Table 1:** Distribution of item responses and reliability of multi-item constructs.

Item	Scale	Percentile			Mean (SD)
		25th	50th	75th	
**Component Experiences** a					
*General*					
1. How easy or hard is it to call this clinic during regular hours and get the answers you need?	1–4^b^	2	3	4	3.0 (1.0)
Think about all the care you got at this clinic in the past 12 months.How would you rate the following?					
2. Ease of getting to clinic	1–5^c^	3	4	5	3.7 (1.1)
3. Parking	1–5^c^	2	3	3	2.7 (1.3)
4. Wait time	1–5^c^	2	3	4	3.0 (1.2)
5. Pharmacy	1–5^c^	3	4	5	3.7 (1.2)
6. Lab	1–5^c^	3	4	5	3.6 (1.3)
7. Social work	1–5^c^	3	4	5	3.9 (1.1)
*Facility*, α = 0.85					
8. Noise level	1–5^c^	3	3	4	3.5 (1.1)
9. Cleanliness and look	1–5^c^	3	4	5	4.0 (0.9)
10. Concern for privacy	1–5^c^	3	4	5	4.0 (1.0)
11. Clinic hours	1–5^c^	3	4	5	3.8 (1.0)
*Staff*, α = 0.91					
12. Courtesy of person making appointment	1–5^c^	3	4	5	4.1 (0.9)
13. Helpfulness of front desk staff	1–5^c^	3	4	5	4.0 (1.0)
14. Courtesy of front desk staff	1–5^c^	3	4	5	4.0 (1.0)
15. Nurse’s concern	1–5^c^	3	4	5	4.0 (1.0)
*Provider*, α = 0.84					
16. Would you recommend your regular HIV provider to other patients with HIV?	1–5^d^	4	5	5	4.6 (0.8)
17. All things considered, how much do you trust your HIV provider?	1–10^e^	9	10	10	9.4 (1.3)
18. Overall, how do you feel about your regular HIV provider?	1–7^f^	6	7	7	6.5 (1.1)
19. If you could switch to another HIV provider at this clinic, would you?	1–5^g^	4	5	5	4.3 (1.1)
**Overall Satisfaction,** α = 0.70					
1. Would you recommend this clinic to other patients with HIV?	1–5^d^	4	5	5	4.6 (0.7)
2. Overall, how do you feel about the care you got at this clinic in the past 12 months?	1–7^f^	6	7	7	6.2 (1.2)
3. If you could switch to another HIV clinic in this area at the same cost, would you?	1–5^g^	3	4	5	4.1 (1.1)

SD indicates standard deviation; α indicates Cronbach’s alpha coefficient.

a In the 12 months prior to survey completion, a total of 36 participants had not called the clinic, 128 participants did not drive to clinic, 90 participants did not use the clinic pharmacy, and 180 participants had not seen the social worker.

b 1 = very hard, 2 = somewhat hard, 3 = somewhat easy, 4 = very easy.

c 1 = poor, 2 = fair, 3 = good, 4 = very good, 5 = excellent.

d 1 = definitely no, 2 = probably no, 3 = not sure, 4 = probably yes, 5 = definitely yes.

e 1 = I do not trust my HIV provider → 10 = I trust my HIV provider completely.

f 1 = completely dissatisfied, 2 = mostly dissatisfied; 3 = somewhat dissatisfied, 4 = mixed feelings, 5 = somewhat satisfied, 6 = mostly satisfied, 7 = completely satisfied.

g 1 = definitely yes, 2 = probably yes, 3 = not sure, 4 = probably no, 5 = definitely no.

The questions about recommendation and trust were adapted from the Ambulatory Care Experiences Survey [21]. The questions about feelings towards the provider and overall care were based on the Delightful–Terrible 7-point scale, a validated measure of life satisfaction [19]. The switching question was based on brand and product switching behavior frequently cited in marketing research [22]. For ease of interpretation, the satisfaction responses were transformed to a 0- to 10-point scale.

Given the time constraints involved with administering surveys in a waiting room setting, we used validated, single-item questions to identify individuals with possible depression, excessive alcohol use, and illegal or prescription drug abuse [23]–[25]. We also used a validated, single item question to measure health status, “In general, how would you rate your overall health?” [26]–[28]. Pearlin’s 7-item Self-Mastery Scale assessed self-efficacy [29]–[31]. Commonly used questions provided data on patient demographics and HIV risk factors [21], [32].

To ensure a comprehensive list of component experiences that affect how patients evaluate their overall care, we performed in-depth interviews with key clinic staff. We interviewed physicians, nurses, administrators, clerical personnel and the physician assistant to gain insight into the various components of the clinic care process. The breadth of component experiences was confirmed during pre-testing of the survey instrument.

With the help of a health communications specialist, we simplified the wording and flow of the survey so that certain patients with low health literacy could complete it with ease [33]. The survey was translated into Spanish and the translation was reviewed by two Spanish speakers. The survey has a Flesch-Kincaid 6^th^ grade reading level and takes about 10 minutes to complete.

### Pre-testing the Survey Instrument

We conducted one-on-one, face-to-face cognitive interviews to pretest the survey instrument and ensure that the survey was appropriately tailored to our population of mostly low-income, ethnic-minority patients living with HIV. These interviews confirmed survey comprehension and conceptual equivalence of the English and Spanish versions of the survey [34]. Finally, we asked open-ended questions about component experiences that shape how patients evaluate their overall care in clinic. This served to verify that the list of component experiences was complete.

Pre-testing continued until redundancy in data was reached. The convenience sample included 11 English- and 10 Spanish-speaking patients. Interviews were audio-taped and lasted about 1 hour. Each participant received $10. We revised the survey as follows, based on results of cognitive interviews: 1) wording of certain survey questions was modified; 2) laboratory and social work services were added as component experiences; and 3) the response scale anchors for the questions about feelings toward the provider and care were changed from “delighted - terrible” to “completely satisfied - completely dissatisfied.”

### Survey Administration

Preliminary study eligibility was determined by reviewing medical records. Employing a systematic sampling method, patients who met the inclusion criteria were approached based on check-in times. When a member of the research team became available to administer the survey, he/she approached the eligible patient with the most recent check-in time. Patients were told that personal data would be kept confidential, and answers would not affect their care. Patients completed the surveys while they waited for their appointment. Survey mode was coded as interviewer-administered if the patient received assistance in completing the survey.

### Clinical and Demographic Characteristics

Electronic medical records and administrative data were reviewed. The following data were abstracted for participants: age, date of initial visit at the clinic, appointment data, CD4 cell count, and HIV viral load. For eligible patients who declined to participate, data abstraction was limited to age, race, sex, and ethnicity.

### Quality Control

A single staff member performed double data entry of completed surveys. Five percent of medical records and administrative data were reviewed manually to make sure the data were abstracted correctly.

### Statistical Analysis

#### Exploratory factor analysis

Exploratory factor analysis of the 15 clinic and staff attributes determined whether certain attributes were similar enough to group into multi-item constructs. We used principle components analysis with the communality estimates set to one. Analysis revealed two interpretable factors, which explained 58% of the variance in these attributes. The factor solutions were rotated using an orthogonal rotation (varimax method) and interpreted using factor loadings of 0.6 or higher and similar thematic content [35]. The first factor reflected evaluations of the facility environment (noise, cleanliness, concern for patient privacy and clinic hours). The second factor reflected evaluations of the staff (person making appointment, front desk staff, and nurse). Based on these findings, facility and staff were defined as separate multi-item constructs. The third factor was not interpretable and the remaining attributes were treated as single-item measures.

Provider satisfaction was distinguished from overall satisfaction with care by performing a principle components analysis of the items measuring these constructs. Analysis revealed two factors, which explained 66% of the variance. The first factor reflected evaluations of the provider (likelihood of recommending provider, trust with provider, feelings about provider, and intention to switch provider). The second factor reflected evaluations of overall satisfaction with care received in the clinic (likelihood of recommending the clinic, feelings about care, and intention to switch clinic). These findings confirmed the treatment of provider and overall satisfaction as separate multi-item constructs.

#### Validity and reliability

The validity of the four multi-item constructs (facility, staff, provider and overall satisfaction) was assessed by examining Pearson’s correlations between the individual items comprising the constructs. The multi-item constructs demonstrated satisfactory levels and patterns of intra-scale correlation (Table 2). High correlations between items intended to measure a given construct suggested the convergent validity of those measures. Likewise, substantially lower correlations between items within a given construct and items intended to measure other constructs suggest the discriminant validity of these measures.

**Table 2:** Item intercorrelations of patients’ component experiences and overall satisfaction.

		Item Intercorrelations										
		1	2	3	4	5	6	7	8	9	10	11
1	Ease of callingclinic	1.00										
2	Ease of gettingto clinic	0.32	1.00									
3	Parking	0.28	0.38	1.00								
4	Wait time	0.38	0.35	0.31	1.00							
5	Pharmacy	0.37	0.39	0.37	0.57	1.00						
6	Lab	0.28	0.34	0.45	0.41	0.60	1.00					
7	Social work	0.38	0.37	0.22	0.48	0.49	0.45	1.00				
8	Facility	0.36	0.48	0.28	0.54	0.60	0.43	0.59	1.00			
9	Staff	0.38	0.54	0.33	0.55	0.59	0.51	0.58	0.72	1.00		
10	Provider	0.22	0.23	0.16	0.26	0.23	0.20	0.20	0.33	0.33	1.00	
11	Overallsatisfaction	0.38	0.31	0.22	0.36	0.41	0.31	0.38	0.51	0.50	0.61	1.00

We tested internal consistency reliability by estimating Cronbach’s alpha coefficient. The Cronbach’s alpha coefficient for the facility, staff, provider and overall satisfaction constructs were 0.85, 0.91, 0.84, and 0.70, respectively (Table 1). Scores for each multi-item construct were obtained by averaging the responses to all items comprising the construct.

#### Bivariate analyses

Bivariate analyses between potential confounders and overall satisfaction with care were performed. Demographic, health status, behavioral characteristics, and clinic utilization listed in Table 3 were included in the regression model of overall satisfaction as potential confounding variables if its bivariate correlation or t-test reached a significance level of p<0.10. We used a significance threshold of p<0.10 instead of p<0.05 to minimize the risk of omitting a potential confounding variable from the final regression.

**Table 3:** Baseline characteristics of participants at Thomas Street Health Center and the Veterans Affairs Medical Center in Houston, Texas (N = 489).

Characteristics	
Age, years – mean (±SD)	48 (±11)
Gender – (%)	
Male	71
Female	29
Race ethnicity – (%)	
Non-Hispanic black	61
Non-Hispanic white	15
Hispanic	21
Other	3
Language preference – (%)	
English	90
Spanish	10
Survey mode – (%)	
Self-administered	85
Interviewer-administered	15
Education – (%)	
Some high school or less	22
High school graduate or equivalent	35
Some college of higher	43
Relationship status – (%)	
Married	14
In a relationship and not married	14
Single	71
Income – (%)	
≤ $10K	54
> $10K and ≤ $30K	36
> $30K	10
Health status – (%)	
Poor/fair	20
Good/very good	65
Excellent	15
Substance use in past year	
Illegal or Rx drug abuse	19
EtOH screen, positive	42
Depression screen, positive	43
Self-efficacy a – mean (±SD)	3.1 (±0.6)
HIV provider visits in past 12 months – mean (±SD)	3.3 (±1.5)
Time enrolled in clinic, years – mean (±SD)	7.6 (±4.6)
HIV risk factor – (%)	
IVDA	16
MSM, no IVDA	33
Heterosexual sex, no IVDA	50
Transfusion	<1
CD4 cell count b – median (25^th^, 75^th^ percentiles)	449 (276, 665)
HIV RNA <48 copies b – (%)	70

SD indicates standard deviation; IVDA intravenous drug abuse; MSM, men who have sex with men;

a Scale 1–4, higher score indicates greater self-efficacy, Cronbach’s α = 0.79.

b Value closest to date of survey completion, ±30 days; CD4 and HIV RNA values available for 84% of participants.

#### Multiple regression analyses

We performed multiple linear regression analyses to determine the strength of association between component experiences and overall satisfaction. Specifically, we estimated two regression models in a hierarchical fashion. Model 1 consists of only control variables and serves to evaluate their predictive ability as a group. Model 2 consists of control variables and the predictors of interest (i.e. component experiences), and serves to show the incremental explanation achieved by the component experiences beyond that of the control variables alone. The key assumptions of multiple regression analysis (i.e. linearity, normal distribution of residuals, constant variance of residuals, and absence of multicollinearity) were tested and verified. Pair-wise deletion of missing data was conducted.

Statistical analyses were performed with SAS version 9.2 (SAS Institute Inc, Cary, NC) and SPSS version 19 (SPSS Inc, Chicago, IL).

The Institutional Review Board for Baylor College of Medicine and Affiliated Institutions approved this study. Participants provided verbal informed consent.

## Results

### Study Population Characteristics

Of 553 patients approached, twenty eight declined, twenty six met the exclusion criteria, four did not finish the survey in the allotted time, and six did not meet the inclusion criteria on further record review. A total of 489 patients were included in the analyses, 101 from VAMC and 388 from TSHC. The participation rate among eligible patients was 94% (489/521). Participants were similar to eligible non-participants in terms of age, race, sex, and ethnicity. As shown in Table 3, a majority of the participants were men (71%), non-Hispanic black (61%), had a household income of ≤ $10,000 (54%), and reported unprotected heterosexual contact as an HIV risk factor (50%). A total of 97% of participants reported having a “regular personal HIV provider.”

### Item Nonresponse

A total of 73.2% of participants had no missing items, and 94.1% had a missing item rate of <5%. The average rate of missing items for a given participant was 1.4%. Rates of nonresponse for individual survey items were low, ranging from 0.2% to 4.1%.

### Ratings of Component Experiences and Overall Satisfaction

Table 1 displays the distribution of patient ratings. The ratings for the component experiences were generally high, with the exception of parking and wait time (mean scores were 2.7 and 3.0, respectively, on a 5.0 scale). Patients reported high levels of overall satisfaction with care received. For example, over 90% of patients stated that they would probably (23.4%) or definitely (69.8%) “recommend this clinic to other patients with HIV,” and over 80% stated that they felt mostly satisfied (26.7%) or completely satisfied (57.3%) with the care they received in clinic.

### Relationship between Component Experiences and Overall Satisfaction

Age, health status, depression, relationship status, education, self-efficacy, Spanish language preference, time enrolled in the clinic and survey mode met the criteria for entry into the multiple regression analysis as control variables. Table 4 shows the linear regression model of patient component experiences on overall satisfaction with care received in clinic. Listwise and mean substitution treatment of missing values yielded comparable results. Results did not differ between the clinic sites.

**Table 4:** Multiple regression of patients’ component experiences on overall satisfaction.

Model 1 (controls only)^a^				Model 2 (controls + component experiences)^b^					
	Standardized β		*P*		Unstandardized B		Standardized β		*P*
Controls				Controls					
Health status	0.190		0.012	Health status	0.096		0.057		0.329
Self-efficacy	0.116		0.138	Self-efficacy	0.069		0.024		0.695
Depression	−0.006		0.935	Depression	−0.107		−0.031		0.594
Relationship status	−0.086		0.214	Relationship status	−0.108		−0.045		0.381
Spanish language preference	0.068		0.385	Spanish language preference	0.047		0.008		0.892
Age	0.129		0.088	Age	0.006		0.039		0.495
Education	−0.091		0.243	Education	−0.114		−0.073		0.220
Survey mode	0.065		0.367	Survey mode	0.315		0.065		0.231
Time enrolled in clinic	0.059		0.417	Time enrolled in clinic	<0.001		0.013		0.813
				Component experiences					
				Provider	0.480		0.445		0.000
				Ease of calling clinic	0.206		0.124		0.038
				Facility	0.359		0.171		0.038
				Staff	0.322		0.161		0.062
				Pharmacy	0.140		0.094		0.232
				Wait time	−0.093		−0.064		0.360
				Social work	0.069		0.044		0.525
				Ease of getting to clinic	−0.056		−0.035		0.595
				Lab	−0.038		−0.028		0.702
				Parking	−0.013		−0.010		0.877

β indicates beta coefficient.

Pairwise approach to data retention.

a Adjusted R^2^ = 0.059; F = 2.397; df 9, 190; p = 0.014.

b Adjusted R^2^ = 0.487; F = 10.925; df 19, 180; p<0.001.

The regression model had an adjusted R square of 0.487 (p<0.001), indicating that the component experiences account for almost half of the explained variation in overall patient satisfaction. The top four component experiences driving overall satisfaction were: 1) satisfaction with the HIV provider (standardized β = 0.445, p<0.001), 2) facility environment (standardized β = 0.171, p = 0.038, 3) ease of calling the clinic and getting answers (standardized β = 0.124, p = 0.038), and 4) staff (standardized β = 0.161, p = 0.062). The large β coefficients reflect a strong relationship between the component experience and overall satisfaction. The size of the unstandardized b coefficient for each component experience indicates the rate of change in overall satisfaction for each unit change in that component experience. For example, satisfaction with the provider has an unstandardized B coefficient of 0.480. This means that a 1-point increase in satisfaction with the provider is associated with 0.480-point gain in overall satisfaction. Patients’ evaluations of wait time, parking, ease of getting to clinic, social work services, pharmacy, and laboratory were not significantly related to overall satisfaction.

## Discussion

In this study of 489 participants receiving outpatient HIV primary care, patients’ evaluation of their provider correlated the strongest with their overall satisfaction and accounted for almost half of the explained variance. Access and availability evaluations, like clinic hours and ease of calling the clinic, also correlated with overall satisfaction, but less strongly. Patients generally gave high overall satisfaction ratings.

The upwardly skewed distribution of patient satisfaction responses is not surprising. Applied customer satisfaction research shows that most people are very satisfied with most products and services they buy [36]. It is the change in the proportion of responses falling into the uppermost response categories (e.g. “top box” scores) over time that provides the most insight into an organization’s performance trajectory [37], [38]. Our findings of high levels of satisfaction are also consistent with other patient satisfaction studies [4], [39], [40]. Interestingly, certain component experiences, like wait time and parking, did not correlate with overall satisfaction. Although patients gave low ratings for wait time and parking, dissatisfaction with those components did not translate into lower levels of overall satisfaction. Our results suggest that as long as patients have positive experiences with their provider, they tend to overlook shortcomings in certain non-interpersonal aspects of care. As such, if clinics want to improve the overall patient care experience, they need to develop strategies to improve patients’ evaluation of their providers.

The facility environment and ease of calling the clinic constructs were also associated with overall satisfaction. The facility construct included questions about the clinic’s concern for patient privacy, clinic hours, noise, and cleanliness. Patients with HIV infection experience actual and perceived HIV-related stigma; thus, privacy concerns may influence their overall satisfaction [41]. Experiences included in the facility and ease of calling the clinic constructs represent modifiable components and, while not major drivers, could serve as easy targets for improvement.

The staff construct (front desk staff and nurse) was associated with overall satisfaction, but did not reach a statistical level of significance. Many studies in the patient satisfaction literature report an association between satisfaction with nursing care and overall satisfaction [42]–[45]. Most of these studies took place in the hospital setting where nursing care occurs continuously. In contrast, patients in the outpatient clinic setting tend to have brief encounters with nurses relative to providers, which may explain our findings.

Limited cross-sectional studies suggest that patients’ evaluation of their provider is important because it impacts subsequent patient behavior, like treatment adherence and intention to return to the provider [46]–[48]. However, it is unclear which aspect of provider care most strongly drives patients’ evaluation. Interpersonal dimensions such as provider warmth, empathy, trust, and communication skills have been associated with more favorable patient evaluations [4], [39], [40]. However, studies consistently show that these factors only explain a small fraction of variance in overall satisfaction scores. Organizational factors beyond a provider’s control may affect patients’ evaluation of their providers. For example, adequate time allotted for clinic visits, continuity of care with the same provider, and minimal wait time between requests for an appointment and the actual appointment date have been associated with favorable patient evaluations [49]–[51]. Additionally, satisfaction with a treatment regimen (e.g. complexity, discomfort, convenience) may also affect how patients rate the provider experience [52]. In-depth qualitative and longitudinal quantitative studies are needed to better understand which provider attributes correlate most highly with patients’ perception of their provider. In addition, longitudinal studies would allow for patient evaluations at multiple points in time and inform how patients’ evaluations of their providers impact subsequent behavior and, ultimately, clinical outcomes.

This study has several methodological strengths. Our study included a predominantly low-income, ethnic-minority population generally not well represented in patient satisfaction studies. Cognitive interviews ensured that survey items reflected all aspects of the clinic experience salient to patients. The study population was systematically sampled, the participation rate was high, and the item non-response rate low. Finally, when possible, we used multi-item constructs to minimize the risk of measurement error.

This study also has certain limitations. The correlational nature of our data precludes causal inferences. The mere participation in a satisfaction survey may inflate respondents’ ratings of satisfaction, a behavior noted in the marketing literature as “the question-behavior effect” [53], [54]. In addition, participants were enrolled in care at the VA and a public clinic, and the findings may not generalize to patients in other settings. Lastly, the rates of depression, excessive alcohol use, and illegal or prescription drug abuse may represent conservative estimates due to the use of single-item screening questions.

### Conclusion

This study quantifies the relative importance of each component experience in shaping patients’ evaluation of their overall satisfaction with care. The findings provide an insight into the component experiences organizations should focus on to most effectively manage patient experiences.

In our study, satisfaction with one’s HIV primary care provider most strongly predicted overall satisfaction with care received in clinic. The patient-provider relationship exceeds other component experiences of care in its association with overall satisfaction. Our study suggests that focusing on improving patients’ satisfaction with their provider yields the highest return on investment, and that interventions to improve overall patient satisfaction should target this dimension of care.

## Acknowledgments

This project was supported in part by the facilities and resources of the Houston Veterans Affairs Health Services Research and Development Center of Excellence, Michael E. DeBakey Veterans Affairs Medical Center and the Harris County Hospital District, Houston, Texas, United States of America. The views expressed in this article are those of the authors and do not necessarily represent the views of the Department of Veterans Affairs.
